# New reliable scoring system, Toyama mouse score, to evaluate locomotor function following spinal cord injury in mice

**DOI:** 10.1186/1756-0500-7-332

**Published:** 2014-06-03

**Authors:** Michiko Shigyo, Norio Tanabe, Tomoharu Kuboyama, Song-Hyen Choi, Chihiro Tohda

**Affiliations:** 1 Division of Neuromedical Science, Institute of Natural Medicine, University of Toyama, 2630 Sugitani, Toyama 930-0194, Japan; 2 Dong-A ST Pharm. Research Center, 47-5 Sanggal-dong, Giheung-gu, Yongin-si, Gyeonggi-do 446-905, Republic of Korea

**Keywords:** Spinal cord injury, Hindlimb movement, Mouse, New scoring, TMS

## Abstract

**Background:**

Among the variety of methods used to evaluate locomotor function following a spinal cord injury (SCI), the Basso Mouse Scale score (BMS) has been widely used for mice. However, the BMS mainly focuses on hindlimb movement rather than on graded changes in body support ability. In addition, some of the scoring methods include double or triple criteria within a single score, which likely leads to an increase in the deviation within the data. Therefore we aimed to establish a new scoring method reliable and easy to perform in mice with SCI.

**Findings:**

Our Toyama Mouse Score (TMS) was established by rearranging and simplifying the BMS score and combining it with the Body Support Scale score (BSS). The TMS reflects changes in both body support ability and hindlimb movement. The definition of single score is made by combing multiple criteria in the BMS. The ambiguity was improved in the TMS. Using contusive SCI mice, hindlimb function was measured using the TMS, BMS and BSS systems. The TMS could distinguish changes in hindlimb movements that were evaluated as the same score by the BMS. An analysis of the coefficient of variation (CV) of score points recorded for 11 days revealed that the CV for the TMS was significantly lower than the CV obtained using the BMS. A variation in intra evaluators was lower in the TMS than in the BMS.

**Conclusion:**

These results suggest that the TMS may be useful as a new reliable method for scoring locomotor function for SCI models.

## Findings

### Background

A variety of methods to evaluate locomotor function following SCI have been proposed and modified over time. Especially, open field locomotion has become a widely used and important means of evaluation for SCI models because it is easily accessible (no required instruments). Among these methods, the Basso-Beattie-Bresnahan scoring (BBB) system [1] has been widely used in the rat model. However, BBB is not as suitable for evaluation in a mouse model. Therefore, Basso et al. developed a new scoring method for mice, the Basso Mouse Scale score (BMS) [2].

In the BMS, score 3 reflects the threshold at which the animal can support its own body weight. The potency of supporting the body trunk is graded according to the functional improvement. However, beyond score 3, stepping frequency and consistency are the primary factors evaluated, suggesting that the BMS fails to detect differences in ability of body support. Therefore, we proposed the Body Support Scale score (BSS) an additional criterion for functional evaluation [3].

Some points of the BMS still should be improved. Double or triple criteria are considered to determine for scores 3, 5, 6, 7 and 8, so that a broad range of functional states may be classified in a single score in BMS. In this study, we propose a new scoring system named the Toyama Mouse Score (TMS) that is based on a combination of the BMS and BSS with modifications. The TMS is easy to access, and offers a clear-cut summative pointing system, resulting in better evaluation with high sensitivity and low variation.

## Methods

### Spinal cord injury

**Figure 1:**
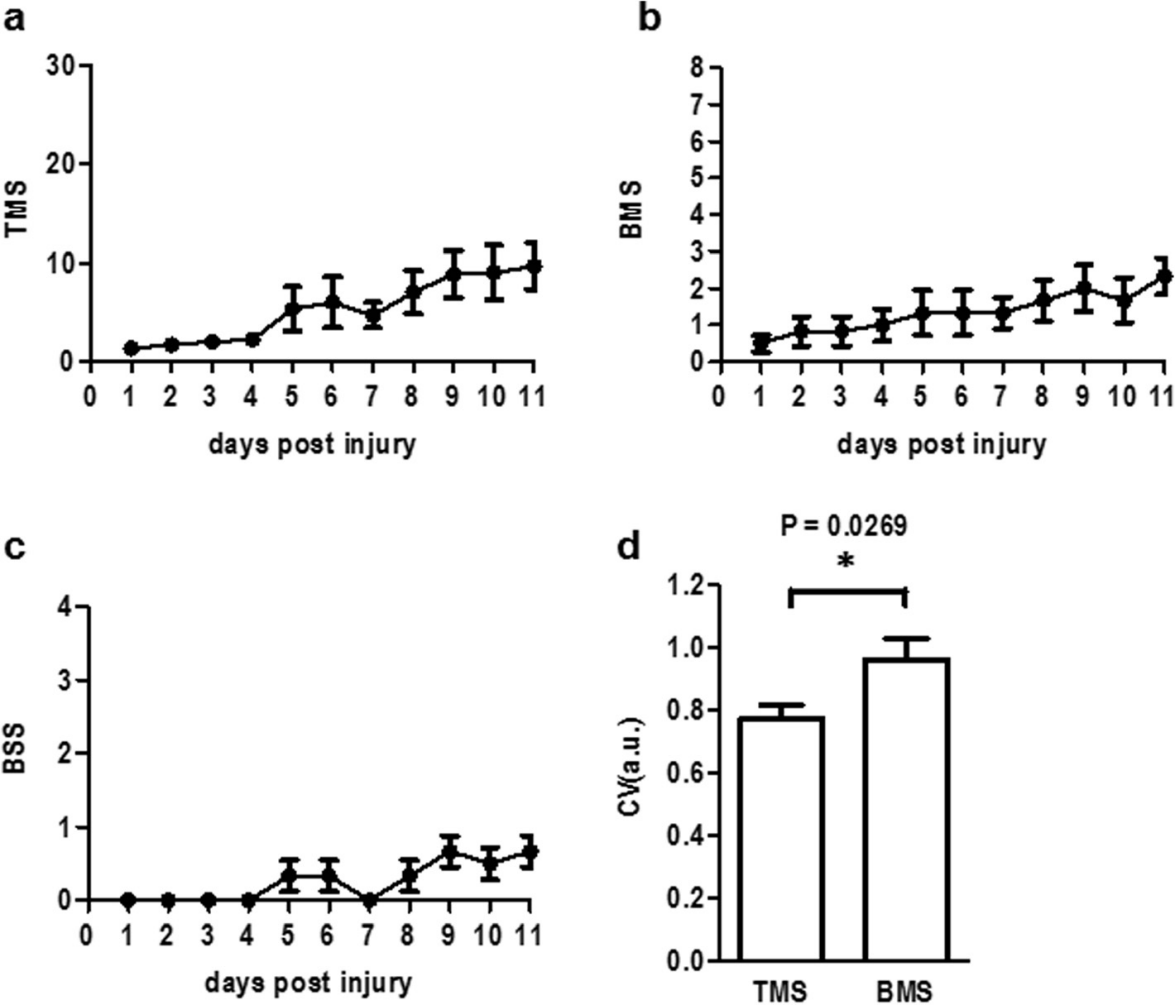
**Hindlimb function in SCI mice evaluated by TMS, BMS and BSS.** The locomotor function of SCI mice was evaluated by using the TMS **(a)**, BMS **(b)** and BSS **(c)** for 11 days post injury (a total of 3 mice per group, n = 6 hindlimbs). Coefficients of variation (CV) for the TMS and BMS were calculated for each of the 11 days **(d)**. The TMS yielded a significantly lower CV than did the BMS (*p* = 0.0269, *p < 0.05, unpaired *t*-test).

The committee for Animal Care and use at the University of Toyama approved each of the study protocols. Six-week-old male (Figure 1, Table 1) and 8-week-old female (Table 2) ddY mice (SLC, Shizuoka, Japan) were used in the SCI experiments. The mice were anesthetized by the administration of trichloroacetaldehyde monohydrate (500 mg kg^-1^, i.p.). After confirming the mice were completely under anesthesia by pinching the hindpaw, the surgical operation to induce SCI was performed, as previously described [4] with slight modifications. After laminectomy at the T11 vertebrae level, contusion injuries were given by dropping a 6.5-g weight (the tip diameter : 1 mm) from a height of 30 mm onto the exposed dura mater of the lumbar spinal cord at the L1 level using stereotaxic instrument (Narishige, Tokyo, Japan). The injured mice hardly move their hindlimbs at 1 day post injury (dpi) and come to recover gradually as time goes by, and we have gotten reproducible data about it [3].

**Table 1:** TMS, BMS and CV values for 6 hindlimbs at 4 days post injury evaluated by single observer

	**TMS**	**BMS**
Hindlimb 1	1	0
Hindlimb 2	1	0
Hindlimb 3	0	0
Hindlimb 4	3	2
Hindlimb 5	4	2
Hindlimb 6	4	2
CV	0.795	1.095

**Table 2:** TMS, BMS and CV values for one hindlimb at 3 days post injury evaluated by 8 observers

	**TMS**	**BMS**
Observer 1	3	1
Observer 2	0	0
Observer 3	1	0
Observer 4	3	1
Observer 5	0	0
Observer 6	1	0
Observer 7	1	0
Observer 8	2	0
CV	0.864	1.852

### Locomotor evaluation

For behavioral scoring, the mice were individually placed in an open-field (42 cm × 48 cm × 15 cm) and observed for 5 min once a day until 11 days post injury. In the control group, naive ddY mice were observed for 5 days. Open field locomotion was evaluated using the 0–8 point BMS score (without tail score), the 0–4 point BSS score and the 0–30 point TMS. Animals were allowed to move freely in the plastic box. The movement of the left and right hindlimbs was evaluated independently.

### Statistical analysis

Statistical tests were performed using GraphPad Prism software (version 5; GraphPad Software, Inc., San Diego, CA). Differences in the scores were analyzed using unpaired *t*-tests. The coefficient of variation (CV) was calculated as follows; CV = standard deviation/mean value. *P* values < 0.05 were considered significant.

## Results

SCI mice were observed in an open field and scored using the TMS (Figure 1a), BMS (Figure 1b) and BSS (Figure 1c) for 11 days after SCI. A TMS point table is shown in Table 3. Categories of criteria for the TMS were determined by according to the BMS subscore [2] with modification. We think that body support ability is very important functional outcome and should be weighted equally to other issues. Therefore, in the TMS, the maximum score for body support was 15 points, and the maximum summed score of other issues was also 15 points. Observations were performed for 9 items, and the points were simply summed. Table 4 shows focused points for observation in the TMS, BMS and BSS. The TMS observes a frequency of ankle movement, movement of knees, thighs and toes, which are not checked in the BMS measurement. To compare the precision of the TMS and BMS, we calculated the coefficients of variations (CV) of the data in the 11-day observation periods. CV values are used to evaluate relative variations of data obtained in different criteria. The averaged CV of the TMS data was significantly lower than that of the BMS scores (*p* = 0.0269) (Figure 1d). We additionally performed two independent series of these experiments. The results consistently indicated that CV of the TMS data was significantly smaller than the CV of the BMS data (experiment 1; CV in TMS = 1.1274, CV in BMS = 1.4269, *p* = 0.0286) (experiment 2; CV in TMS = 0.7627, CV in BMS = 1.3560, *p* = 0.0399). To compare the sensitivity between the TMS and BMS, data of 6 hindlimbs at 4 days post injury were picked up from Figure 1 (Table 1). As a result by single observer, three hindlimbs evaluated similarly as score 0 by the BMS were distinguish as point 0 or 1 in the TMS. Also, three hindlimbs evaluated as score 2 by the BMS were judged as points 3 or 4 in the TMS. The CV value of the TMS was also lower than that of the BMS at the point. These results suggest the fine sensitivity of the TMS. In an independent experiment, score points observed by different observers for one hindlimb at 3 days post injury were picked up to compare variations in intra evaluators between the TMS and BMS (Table 2). The results showed low CV value of the TMS compared with CV of the BMS, suggesting that TMS provides lower variation in intra observers than BMS. A similar tendency was observed also in other mice. Naive mice without injury were observed for 5 days by 6 observers. The TMS, BMS and BSS were shown in full scores by all observers. These results suggest that the TMS provides reliable data with little deviation.

**Table 3:** TMS point table for the open field locomotor performance of SCI mice

**Ankle movement**				**Movement in other joints**^**3)**^		**Toe movement**^**4)**^		**Touchable area of the sole**				**Coordination**^**5)**^		**Hindlimb movement at stepping**		**Body supporting**	
**Frequency**^**1)**^		**Mobile extent**^**2)**^						**At resting**		**At stepping**							
No (0)	0	No (0)	0	No	0	No	0	No	0	No	0	No	0	No	0	No	0
<50%	1	<50%	1	Yes	1	Yes	1	Partial sole touch	1	Partial sole touch	1	Yes	1	Rotative	1	Sometimes support of hind body trunk	5
≥50%	2	≥50%	2					Full sole touch	2	Full sole touch, frequency <50%	2			Parallel	2	Always support of the body trunk, but unstable weight support	10
										Full sole touch, frequency ≥ 50%	3					Always support of the body trunk, and stable weight support	15
										Full sole tohch in every steps	4						

Definitions

^1)^Frequency: a ratio of the number of stepping forward with the ankle movement in all steppings.

^2)^Mobile extent: Mobile extent observed in a normal mouse is defined as 100%.

^3)^Movement of the knee joint or hip joint.

^4)^Toe movement: Toes movement, but not spasm.

^5)^Coordination: Correspondence between the forelimbs and hindlimbs in steppings.

**Table 4:** Focused points in TMS, BMS and BSS

**Criteria**		**TMS**	**BMS**	**BSS**
Ankle movement	Frequency	G	-	-
	Mobile extent	G	G	-
Movement in other joints		Y / N	-	-
Toe movement		Y / N	-	-
Touchable area of the sole	at Resting	G	Y / N	Y / N
	at Stepping	G	G	-
Coordination		Y / N	G	-
Hindlimb movement at stepping		G	G	-
Body supporting		G	Y / N	G

Y / N: Score points are judged in yes or no.

G: Score points are judged with several grades of functions.

-: Not determined.

## Discussion

We established a new method to evaluate SCI mice, the TMS by rearranging and simplifying the BMS and combining it with the BSS. The TMS provided a low coefficient of variation relative to the BMS in SCI mice. The decision for each factor on the TMS is easy because the criteria are clear. In contrast, the BMS yielded larger deviations than the TMS did in intra samples (Figure 1d and Table 1) and in intra observers (Table 2). This larger deviation denotes that scores both higher and lower than the actual value are liable to arise when using the BMS. The TMS reflects changes in both hindlimb movement and body support ability. Since the ambiguous combination of double or triple criteria within a single score is used to judge in the BMS, the ambiguity was improved in the TMS. The TMS also could distinguish slight differences in hindlimb function, which are not detected in the BMS, especially when the score is low (Table 1 and Table 2). The summation system of the TMS does not miss the movement of multiple joints in hindlimbs and toes, and the paw position at resting as well as at walking, resulting in detection of slight changes.

Other groups have also reported improved scoring methods for evaluating SCI mice. The mBBB is an optimized version of the BBB that has been adapted to mice by combining BBB with a walking test on the bar [5], but its use has not spread. The probable drawback to this method is that it is time consuming to perform a walking test. The BLG score [6] was developed by combining the BMS-, with the ladder and grip tests. Here, several instruments are needed to evaluate each criterion. In contrast, the TMS requires only an open box to evaluate SCI mice, making the present system very simple to perform. Our findings suggest that the TMS should be useful as a new reliable scoring system for evaluating SCI mice.

## Competing interests

The authors declare that they have no competing interest related to this study.

## Author’s contributions

MS, NT and TK decided the criteria of TMS. NT and SC performed SCI surgery and observation. MS and NT analyzed the data. TK and CT conducted the experiment. MS and CT wrote the manuscript. All authors approved the final manuscript.
